# *O*-mannosylation and *N*-glycosylation: two coordinated mechanisms regulating the tumour suppressor functions of E-cadherin in cancer

**DOI:** 10.18632/oncotarget.11245

**Published:** 2016-08-12

**Authors:** Sandra Carvalho, Tiago Oliveira, Markus F. Bartels, Eiji Miyoshi, Michael Pierce, Naoyuki Taniguchi, Fátima Carneiro, Raquel Seruca, Celso A. Reis, Sabine Strahl, Salomé S. Pinho

**Affiliations:** ^1^ Instituto de Investigação e Inovação em Saúde (I3S) / Institute of Molecular Pathology and Immunology of the University of Porto (IPATIMUP), 4200-465 Porto, Portugal; ^2^ Institute of Biomedical Sciences of Abel Salazar (ICBAS), University of Porto, 4050-313 Porto, Portugal; ^3^ Centre for Organismal Studies (COS) Heidelberg, Cell Chemistry, University of Heidelberg, 69120 Heidelberg, Germany; ^4^ Department of Molecular Biochemistry and Clinical Investigation, Osaka University Graduate School of Medicine, 565-0871 Osaka, Japan; ^5^ Complex Carbohydrate Research Center, Department of Biochemistry and Molecular Biology, University of Georgia, Athens, GA 30602, USA; ^6^ Department of Biochemistry, Graduate School of Medicine, Osaka University, 565-0871 Osaka, Japan; ^7^ Medical Faculty, University of Porto, 4200-319 Porto, Portugal; ^8^ Department of Pathology, Hospital S. Joao, 4200-319 Porto, Portugal

**Keywords:** E-cadherin, *O*-mannosylation, *N*-glycosylation, gastric cancer

## Abstract

Dysregulation of tumor suppressor protein E-cadherin is an early molecular event in cancer. *O*-mannosylation profile of E-cadherin is a newly-described post-translational modification crucial for its adhesive functions in homeostasis. However, the role of *O*-mannosyl glycans in E-cadherin-mediated cell adhesion in cancer and their interplay with *N*-glycans remains largely unknown. We herein demonstrated that human gastric carcinomas exhibiting a non-functional E-cadherin display a reduced expression of *O*-mannosyl glycans concomitantly with increased modification with branched complex *N*-glycans. Accordingly, overexpression of *MGAT5*-mediated branched *N*-glycans both in gastric cancer cells and transgenic mice models led to a significant decrease of *O*-mannosyl glycans attached to E-cadherin that was associated with impairment of its tumour suppressive functions. Importantly, overexpression of protein *O*-mannosyltransferase 2 (*POMT2*) induced a reduced expression of branched *N*-glycans which led to a protective effect of E-cadherin biological functions. Overall, our results reveal a newly identified mechanism of (dys)regulation of E-cadherin that occur through the interplay between *O*-mannosylation and *N*-glycosylation pathway.

## INTRODUCTION

Epithelial cadherin (E-cadherin) is a cell surface glycoprotein with key roles in normal epithelial homeostasis, cell polarity and tissue architecture [1–3]. E-cadherin extracellular domain mediates homophilic interactions between neighbouring cells [4, 5] while the E-cadherin cytoplasmic domain binds to cytosolic catenins, namely β-catenin and p-120 catenin, providing anchorage to the actin cytoskeleton and contributing to the establishment of stable and mature adhesion complexes- adherens junctions [6, 7]. Reduced expression of E-cadherin and impairment of its functions are well-established molecular events that occur during tumour development and progression, leading to an increased ability of cells to invade surrounding tissues and to metastasize [8–10].

Aberrant glycosylation occurs in essentially all types of human cancers, and thus numerous glycans epitopes constitute tumour-associated antigens [11]. Posttranslational modifications of E-cadherin through glycosylation play an instrumental role in the dysregulation of E-cadherin functions in a cancer context [12–14]. As example, O-linked β-*N*-acetylglucosamine (O-GlcNAc) modification of the E-cadherin cytoplasmic domain was described to retain E-cadherin in the endoplasmic reticulum (ER) inhibiting its trafficking to the plasma membrane, and resulting in reduced intercellular adhesion [15, 16]. An *O*-N-acetylgalactosamine (O-GalNAc) glycosite on EC1 ecdomain of E-cadherin was additionally reported [17]. In fact, altered pattern of protein glycosylation results in the production of different E-cadherin glycoforms that are associated with impairment of its crucial functions in gastric cancer [18].

Modification of E-cadherin with β1,6 GlcNAc branched *N*-glycans, induced by the N-acetylglucosaminyltransferase V (GnT-V), has deleterious effects on E-cadherin-mediated cell adhesion leading to the impairment of the stability and competence of the intercellular adhesive complex [18–20]. The importance of this specific E-cadherin *N*-glycoform was demonstrated to be associated with the invasive and metastatic potential of gastric carcinoma [18]. Moreover, it was recently reported that among the four potential *N*-glycosylation sites on the extracellular domain of E-cadherin, Asn-554 is the key *N*-glycosylation site that, within a gastric cancer context, is selectively modified with the deleterious β1,6 GlcNAc branched *N*-glycans, and directly affects E-cadherin functions [21]. Importantly, prevention of E-cadherin aberrant glycosylation at Asn-554 was found to preclude its functional dysregulation in gastric cancer cell models by improving its cell adhesion functions [21].

Protein *O*-mannosylation is a posttranslational process initiated at the ER by the covalent attachment of mannose structures to serine (Ser) or threonine (Thr) amino acids of secretory and/or membrane proteins, catalysed by the homologous protein O-mannosyltransferases 1 (POMT1) and 2 (POMT2) [22–24]. The mannose residue linked to Ser/Thr may be then further extended with different carbohydrate moieties originating distinct O-mannosyl oligosaccharides [25]. N-acetylglycosaminyltransferase IX (GnT-IX or GnT-Vb [26]) has been identified to be involved in further extension of the O-mannosyl glycans core structures after POMGnT1 activity in α-dystroglycan (α-DG) glycoprotein in the brain [27, 28]. α-DG is an integral glycoprotein of the dystrophin-glycoprotein complex that undergoes O-mannose and mucin-type O-glycosylation [29]. The α-DG links the extracellular matrix (ECM) to the actin cytoskeleton by interacting with ECM proteins in a glycan-dependent manner [30, 31]. Disruption of the *O*-mannosylation pathway, and thus the hypoglycosylation of α-DG result in the impairment of α-DG-mediated epithelial cell-basement membrane interaction [32], and underlies various forms of congenital muscular dystrophies (CMD) [33, 34].

A wide spectrum of other *O*-mannosylated proteins has been recently identified, and among them, cadherins were shown as major *O*-mannosylated glycoproteins [35, 36]. Vester-Christensen et al. identified nine *O*-Man glycosites located on EC2- EC4 ectodomains of E-cadherin [35] being some of these *O*-Man glycosites also found in the crystal structure of E-cadherin [37]. Furthermore, Lommel et al proved directly the presence of an *O*-linked mannose on ectodomain EC4 of E-cadherin that is not elongated further [36]. Defective *O*-mannosylation has been reported to affect the formation of adherens junctions between blastocysts of the mouse embryo and thus required for the morula to blastocyst transition before implantation [36]. Moreover, *O*-mannosylation was found to be important for E-cadherin-mediated cellular adhesion in non-cancer cellular models [36]. Taking into consideration the instrumental role of glycosylation as a mechanism for regulating E-cadherin functions in cancer [12, 13], it is of utmost importance to understand the yet uncovered molecular role of *O*-mannosyl glycans and their interplay with *N*-glycans in the regulation of E-cadherin in the context of cancer.

In this study, we originally report that *O*-mannosylation is overall reduced in human gastric cancer when compared with normal gastric mucosa, by affecting the capacity of E-cadherin to establish competent adhesive complexes. Furthermore, we also demonstrate the existence of a coordinated interplay between complex *N*-glycans and *O*-mannosyl glycans on E-cadherin with biological relevance in a cancer context. Interestingly, preventing the addition of β1,6-GlcNAc-branched *N*-glycans at the E-cadherin Asn-554 promotes its modification with *O*-mannosyl glycans, leading to a recovery of E-cadherin functions. Further, this interplay between branched *N*-glycans and *O*-mannosyl glycans was found to occur *in vivo* using human primary gastric carcinomas and experimental animal models.

## RESULTS

### Protein *O*-mannosylation is reduced in primary human gastric carcinomas

**Figure 1:**
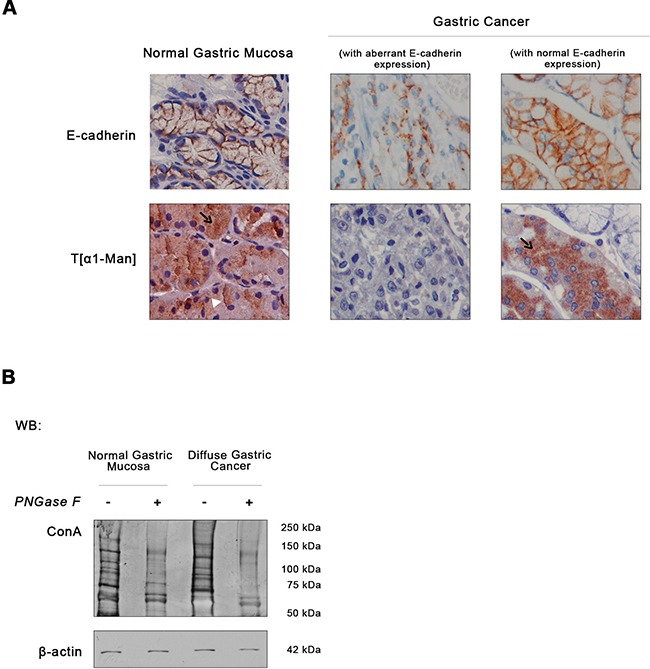
Expression of E-cadherin and *O*-mannosyl glycans in human normal gastric mucosa and diffuse gastric cancer **A.** Immunodetection of E-cadherin and *O*-mannosylated proteins. Immunohistochemical analysis of the normal gastric mucosa (*n*= 10) showed membrane E-cadherin expression, as expected. T[α1-Man] immunohistochemistry detecting threonine *O*-mannosyl-conjugated epitopes revealed a strong intracellular staining of *O*-mannosylated proteins (black arrow) and at the cell membrane (white arrowhead) in the body region of the normal gastric mucosa. Almost no reactivity to T[α1-Man] mAb in neoplastic cells displaying an aberrant expression of E-cadherin at the cytoplasm was observed (*n*= 18). However, intact glands of adjacent non-neoplastic mucosa with membrane E-cadherin expression exhibited intracellular staining of *O*-mannosylated proteins, although less intense than normal gastric body mucosa. Original magnification: 600x. **B.** Evaluation of *O*-mannosyl glycan levels in total lysates from normal gastric mucosa and diffuse gastric carcinoma tissues by lectin blotting. To evaluate the expression of *O*-mannosyl glycans, a blotting using Con A mannose-binding lectin was performed. Con A lectin recognizes α-linked mannose residues from *O*-mannosylated proteins and *N*-glycoproteins. So, tissue lysates were treated with *PNGase F* to remove all types of *N*-glycans before the lectin blotting (see Material and methods' section). As expected, *PNGase F* treatment resulted in a decreased reactivity to the Con A lectin. Regarding the expression of *O*-mannosyl glycans (detected by treatment with *PNGase F* + Con A lectin blotting), diffuse gastric carcinoma displayed a decreased *O*-mannosylation profile. Blot represents two independent biological experiments. Each experiment was performed from the total lysates pooled from different patients and then analyzed as one sample. The same procedure was performed in the second experiment. WB, Western blot.

We sought to understand whether the dysregulation of *O*-mannosylation process is an important oncogenic event in diffuse gastric cancer. We used a unique cohort of well-characterized human primary diffuse gastric carcinomas from the pathology department of Hospital São João, Porto, Portugal, considered an international reference in gastropathology. The expression of *O*-mannosylated proteins was assessed in 18 well-characterized human primary diffuse gastric carcinomas displaying aberrant E-cadherin expression (not due to genetic/structural alterations). Such analysis was performed by using a rabbit monoclonal antibody (mAb) generated to direct against a threonine *O*-mannosyl-conjugated epitope and specifically detects *O*-mannosylated proteins (unpublished data) [36]. Normal gastric mucosa, displaying a normal basolateral cell membrane localization of E-cadherin, exhibited a strong reactivity to T[α1-Man] mAb both at the cytoplasm (staining the ER and Golgi secretory pathway) and cell membrane (Figure 1A). Conversely, in gastric cancer cases showing an abnormal pattern of E-cadherin expression, the levels of *O*-mannosylated glycoproteins decreased significantly. Interestingly, threonine *O*-mannosyl-conjugated epitopes were detected in adjacent non-neoplastic mucosa, predominantly in cells displaying a cell membrane localization of E-cadherin (Figure 1A). Our results point toward a strong association between increased protein *O*-mannosylation in normal/homeostatic conditions, that is significantly reduced in diffuse gastric carcinomas with aberrant expression of E-cadherin (*p value* 7.34 × 10^−6^, Table 1).

**Table 1:** Expression of O-mannosylated proteins in healthy and diffuse gastric cancer patients

	O-mannosylated protein expression			*P value*
	*Negative*	*Low (< 20%)*	*High (> 20%)*	
	*No of patients (%)*			
Normal gastric mucosa	0/10 (0%)	2/10 (20%)	8/10 (80%)	7.34 × 10^−6***^
Diffuse gastric cancer	16/18 (89%)	2/18 (0.11%)	0/18 (0%)	

We also evaluated the *O*-mannosyl glycan profile of total tissue lysates from normal gastric mucosa and gastric carcinoma by using the Concanavalin A (Con A) lectin after previous removal of all type of *N*-linked glycans (high mannose, hybrid and complex type) by *PNGase F* treatment. As shown in Figure 1B, the overall *O*-mannosylation profile is decreased in human primary gastric cancer samples. These observations suggest that primary gastric carcinoma displays low levels of *O*-mannosylated glycoproteins.

### Aberrant E-cadherin expression associated with decreased *O*-mannosylation in cancer

**Figure 2:**
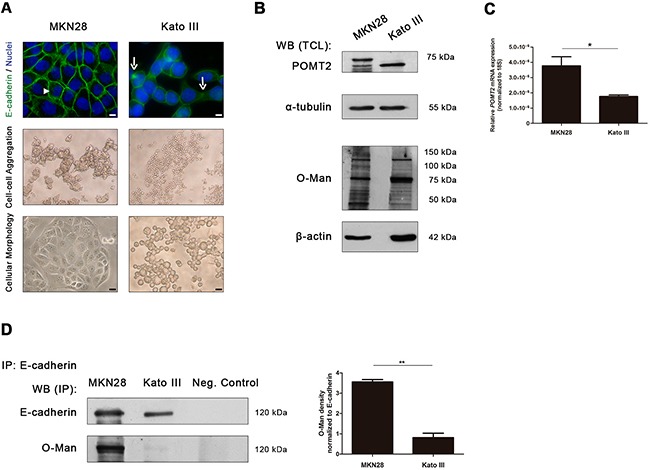
E-cadherin *O*-mannosylation decreases in undifferentiated gastric cancer cells **A.** E-cadherin localization, cell aggregation capacity and morphology in MKN28 and Kato III cell lines. MKN28 cells are characterized by a functional E-cadherin expression at the cell membrane (arrowhead), a high capacity to form large compact cell aggregates and an epithelial cell morphology. In contrast, Kato III cells displayed a reduced expression of E-cadherin at the cell-cell contacts with some mislocalization into the cytoplasm (arrow), decreased cell aggregation capacity and a sparse and rounded morphology. White size bars indicates 5μm. **B.** Evaluation of POMT2 protein and *O*-mannosyl glycans expression in the total cell lysates from MKN28 and Kato III cells by Western blot and Con A lectin staining (which recognizes *O*-mannosyl glycans after remove of *N*-glycans by *PNGase F* digestion), respectively. A decrease of POMT2 protein expression and a distinct *O*-mannosyl glycan pattern was observed in total cell lysate from Kato III, when compared with MKN28. WB (TCL), Western blot (total cell lysate). **C.** *POMT2* mRNA expression. The amount of *POMT2* transcripts was significantly decreased in Kato III cells (*p value*= 0.0293). Plot represents three independent biological experiments. **D.** E-cadherin *O*-mannosylation decreases in undifferentiated gastric cancer cell line. E-cadherin immunoprecipitated from Kato III cells showed decreased Con A lectin reactivity, after removal of *N*-linked glycans by *PNGase F* digestion, when compared with E-cadherin immunoprecipitated from MKN28 cells (4.4-fold; *p value*= 0.0081). Bar graphs, amounts of *O*-mannosyl glycans on E-cadherin are shown as the ratio of densities of Con A lectin and E-cadherin protein reactivity. Results are described as mean ± s.d of three independent biological experiments. IP, immunoprecipitate.

To further evaluate the impact of *O*-mannosylation in E-cadherin, we investigated the expression of *O*-mannosyl glycans in two distinct gastric cancer cell lines with different E-cadherin expression patterns: MKN28 gastric cancer cell line which display normal E-cadherin expression, and Kato III gastric cancer cell line which exhibit an abnormal E-cadherin expression [38–40]. According to Figure 2A, MKN28 cells exhibited a strong membranous staining of E-cadherin (upper panel), high capacity to form large compact cellular aggregates (medial panel) and an epithelial cell morphology (lower panel). In contrast, Kato III cell line is characterized by a reduced E-cadherin expression with some mislocalization into the cytoplasm, reduced cell-cell aggregation capability and a sparsely and rounded cellular morphology.

Concerning POMT2 expression levels, a decrease in the steady-state protein level of POMT2 was observed in Kato III cells when compared to MKN28 cells (Figure 2B). Concordantly, lower levels of *POMT2* mRNA transcripts were detected in Kato III cells (Figure 2C). No significant differences were observed for *POMT1* transcription levels (Supplementary Figure S1A). In addition, the undifferentiated gastric cancer cell line Kato III exhibited a distinct pattern of *O*-mannosylation (evaluated by Con A lectin blotting after removal of all type of *N*-glycans by *PNGase F* digestion) when compared with the well-differentiated MKN28 cells (Figure 2B, Supplementary Figure S1B).

Having demonstrated an association between decreased POMT2 expression (at protein and transcript level) and a distinct pattern of *O*-mannosyl glycans in gastric cancer cells displaying different E-cadherin expression patterns, we further evaluated the expression of *O*-mannosyl glycans specifically on the E-cadherin protein. As depicted in Figure 2D, E-cadherin from MKN28 cells showed a significantly increased reactivity to the Con A- mannose binding lectin (after release of *N*-glycans), when compared with E-cadherin from Kato III (Supplementary Figure S1C). The expression of other glycosylation types, such as *O*-GlcNAc and mucin-type *O*-glycosylation (Sialyl Tn) on E-cadherin in these two gastric cancer cell lines are represented in Supplementary Figure S1C-S1D.

These results strongly indicate that functional E-cadherin (expressed at the cell membrane) is highly O-mannosylated whereas aberrant E-cadherin displays low levels of *O*-mannosyl glycans.

### *POMT2* expression impacts *O*-mannosylation of E-cadherin regulating its biological functions in cancer

**Figure 3:**
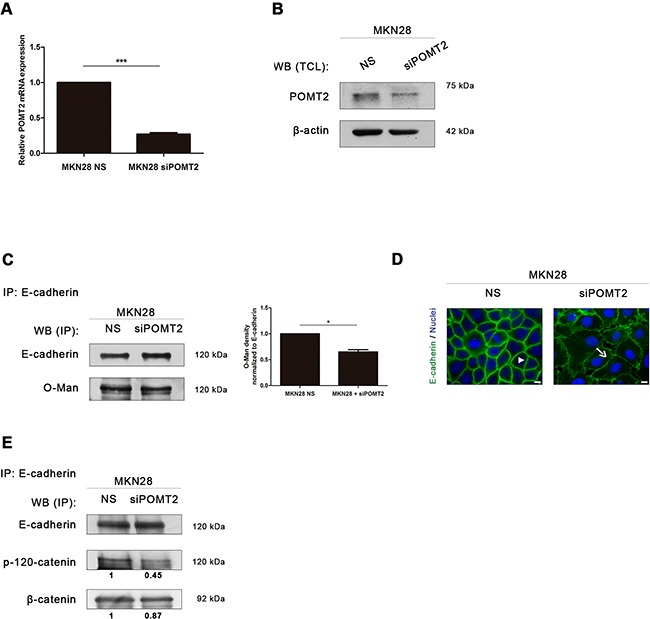
*POMT2* knockdown impairs membrane E-cadherin localization and the assembly of the E-cadherin-catenin complex **A.** *POMT2* mRNA expression in MKN28 cells after *POMT2* knockdown by the small interfering RNA (siRNA) technique. Around 75% of *POMT2* silencing was observed (*p value* < 0.0001). NS, non-silencing. The relative *POMT2* mRNA expression of siPOMT2 cells is shown as the fold increase, compared with NS cells, which was taken as 1. Plot represents three independent biological replicates. **B.** POMT2 protein expression. *POMT2* silencing resulted in a significant decreased expression of POMT2. **C.** *POMT2* silencing resulted in a minor decreased Con A reactivity of the E-cadherin band after removal of *N*-glycans. Bar graphs, amounts of *O*-mannosyl glycans on E-cadherin were determined from the ratios of densities of lectin reactivity (with *PNGase F* treatment) after normalization (as in Figure 2D). Results are described as mean ± s.d of two independent experiments. *P value* = 0.05. **D.** Immunofluorescence analysis of MKN28 cells after *POMT2* silencing demonstrated a reduced E-cadherin localization at the cell membrane and altered cell morphology. White size bars indicate 5μm. **E.** E-cadherin immunoprecipitation after *POMT2* silencing. The co-immunoprecipitated proteins p-120-catenin and β-catenin were identified by western-blot. Knockdown of *POMT2* resulted in a decreased interaction between E-cadherin and p-120-catenin (of about 0.55-fold). No significant differences were observed regarding the interaction of E-cadherin with β-catenin. Amount of association with p-120-catenin and β-catenin were determined as the fold increase, compared with NS cells (which was taken as 1), after normalization to E-cadherin.

**Figure 4:**
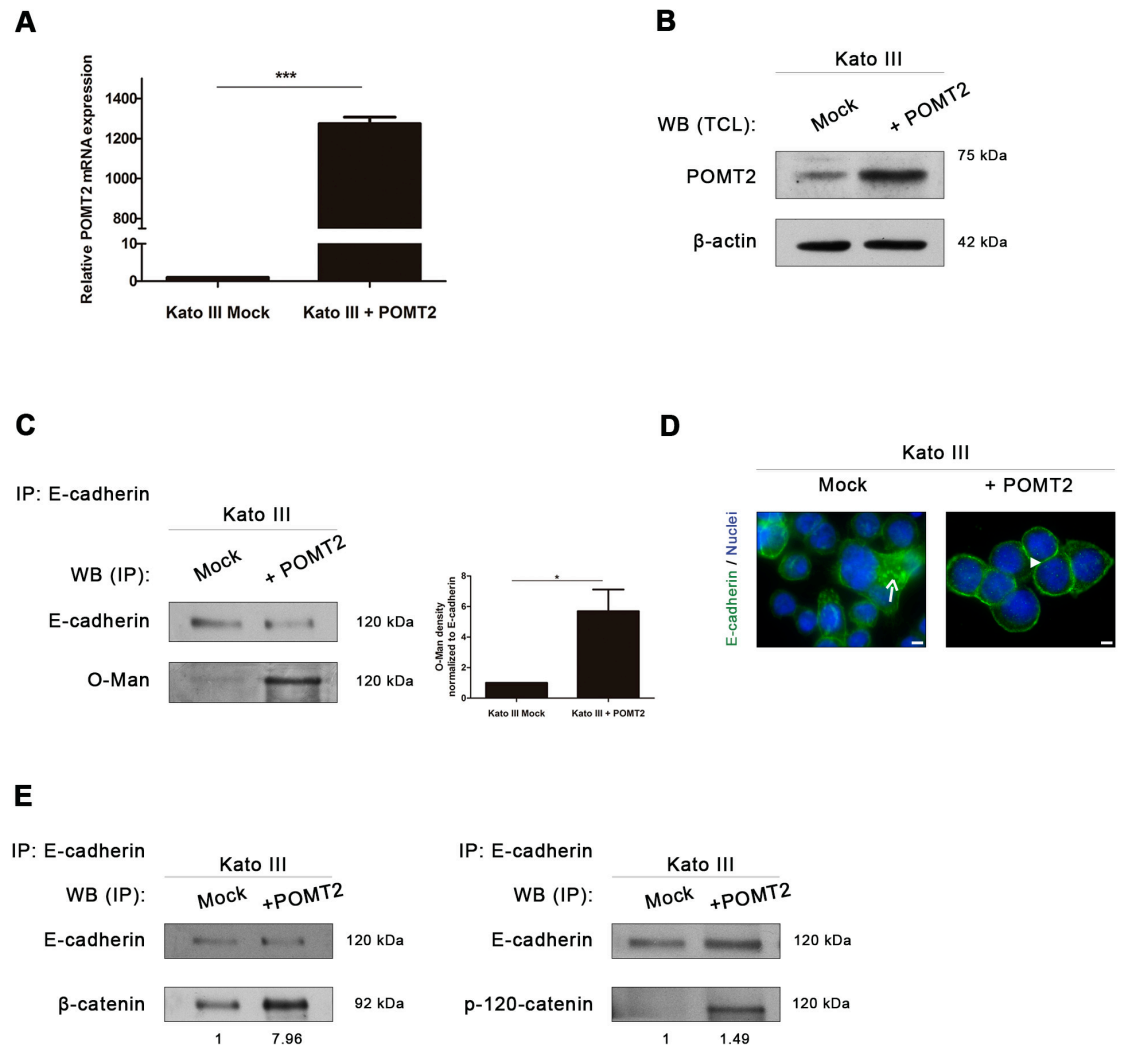
*POMT2* overexpression induces the recovery of E-cadherin biological functions **A.** Confirmation of the overexpressed *POMT2* mRNA transcripts in Kato III cells. The relative *POMT2* mRNA expression is indicated as the fold increase, compared with mock cells, which was taken as 1. Plot represents three independent biological replicates. *P value*< 0.0001. **B.** Overexpression of *POMT2* increases the expression of POMT2 protein. **C.** Evaluation of the impact of *POMT2* overexpression on E-cadherin *O*-mannosylation. *POMT2* overexpression led to a significant increased reactivity of immunoprecipitated E-cadherin to the Con A lectin (after *PNGase F* treatment), suggesting an increase of E-cadherin *O*-mannosylation (around 5.7- fold). Bar graphs, amounts of *O*-mannosyl glycans on E-cadherin were determined as in Fig. 2D. Results are described as mean ± s.d of two independent experiments. *P value* = 0.0376 **D.** Immunofluorescence analysis of Kato III cells overexpressing *POMT2* demonstrated an increased localization of E-cadherin at cell-cell contact sites. White size bars correspond to 5μm. **E.** Evaluation of β-catenin and p-120-catenin recruitment by E-cadherin after *POMT2* overexpression. Kato III cells displayed an increased interaction between E-cadherin and β-catenin and p-120-catenin (around 8.0-fold and 1.5-fold, respectively).

We further evaluated the impact of *O*-mannosylation on the pattern of E-cadherin expression and its molecular interaction with its cytoplasmic partners by silencing *POMT2* in MKN28 cells (Figure 3) or by overexpressing *POMT2* in Kato III cells (Figure 4).

*POMT2* silencing in MKN28 cell line resulted in a significant reduction of *POMT2* mRNA transcripts and decreased POMT2 protein levels (Figure 3A–3B). The impact of *POMT2* knockdown on E-cadherin *O*-mannosylation is not as remarkable as expected (Figure 3C) (*p value* = 0.05), which may suggest other compensatory mechanisms on the process of *O*-mannosylation of E-cadherin. Interestingly, *POMT2* knockdown resulted in an abnormal E-cadherin localization at the cell membrane with a punctuated membrane staining and altered cell morphology (Figure 3D). We further evaluated the impact of decreased *O*-mannosylation in the assembly and stability of the cadherin-catenin complex. The results showed that *POMT2* knockdown led to a significant decreased interaction of E-cadherin with p120-catenin (Figure 3E). No significant differences were observed regarding the recruitment of β-catenin by E-cadherin upon *POMT2* silencing. Overall, these results indicate that *POMT2* knockdown impairs the E-cadherin membrane stability, and disturbs the assembly of the adherens junctions through a decreased recruitment of p-120 catenin by E-cadherin.

Overexpression of *POMT2* in the undifferentiated Kato III cells (confirmed at mRNA and protein levels- Figure 4A-4B) led to a significant increased modification of E-cadherin with O-mannosyl glycans (Figure 4C). Furthermore, the increased E-cadherin *O*-mannosylation was accompanied with a E-cadherin expression at the cell-cell borders (Figure 4D), and increased binding of E-cadherin to β-catenin and p-120-catenin (Figure 4E). These results suggest that *O*-mannosylation of E-cadherin is essential for its correct localization at the cell membrane and contributes for the stability of the adherens junctions in a gastric cancer context.

### *N*-glycosylation and *O*-mannosylation processes as inter-players in the regulation of E-cadherin functions in cancer

Our previous studies demonstrated that addition of GnT-V-mediated β1,6 GlcNAc branched *N*-glycans on E-cadherin contributed to the abrogation of its functions in cancer [18]. Furthermore, E-cadherin was shown to be a target of *O*-mannosylation being important for cadherin-mediated cell adhesion [36]. Taking into consideration these previous observations, we went to evaluate the interplay between these two major forms of E-cadherin modifications: GnT-V-mediated *N*-glycosylation and *O*-mannosylation.

**Figure 5:**
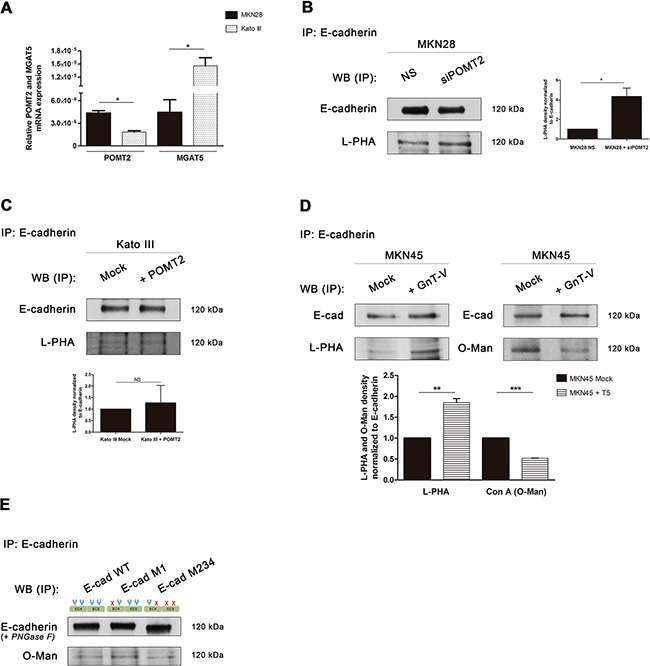
Interplay between GnT-V-mediated *N*-glycosylation and POMT2-mediated *O*-mannosylation of E-cadherin *in vitro* **A.** Evaluation of mRNA transcripts of *POMT2* (*p value* = 0.011) and *MGAT5* (*p value* = 0.0294) in MKN28 versus Kato III cells. Plot represents three independent biological replicates. **B-D.** Reactivity of E-cadherin immunoprecipitated from the different context of *POMT2* and *MGAT5* modulation to the lectins Con A (after removal of *N*-glycans) and L-PHA. Knockdown of *POMT2* **(B)** resulted in increased levels of β1,6 GlcNAc branched *N*-glycans on E-cadherin (around 4.0-fold; *p value* =0.0250), whereas *POMT2* overexpression **(C)** did not alter the reactivity of E-cadherin to L-PHA lectin. **(D)** Overexpression of *MGAT5* in MKN45 cells resulted in an increased L-PHA lectin staining (1.85-fold; *p value* = 0.006) and a decreased reactivity to Con A lectin after *PNGase F* treatment (0.52-fold; *p value* < 0.0001). Bar graphs, amounts of β1,6 GlcNAc branched *N*-glycans and *O*-mannosyl glycans on E-cadherin were determined from the ratios of densities of L-PHA and Con A lectins reactivity respectively, after normalization to E-cadherin. **E.** Evaluation of E-cadherin *O*-mannosylation regarding site-specific *N*-glycosylation occupancy. E-cadherin M1 immunoprecipitated exhibited an increased reactivity to Con A lectin (after *PNGase F* treatment) when compared with E-cadherin WT and mutant M234. Removal of *N*-glycans by *PNGase F* treatment lead to a lower mobility shift of E-cadherin WT and M1 band comparing with E-cadherin M234, which indicates that these E-cadherin *N*-glycoforms may undergo further modification while E-cadherin M234 does not.

The analysis of the transcript levels of *MGAT5* and *POMT2* in these two distinct gastric cancer cell lines showed an inverse relationship: the decreased *POMT2* expression in the undifferentiated gastric cancer cells is accompanied with increased *MGAT5* transcript levels, compared with the well-differentiated MKN28 cells that revealed the opposite (Figure 5A). The analysis of the expression of β1,6 GlcNAc branched *N*-glycans (detected by lectin phytohemagglutinin- L-PHA), and *O*-mannosyl glycans (evaluated by Con A lectin after removal of *N*-linked glycans) on E-cadherin showed that E-cadherin from MKN28 is modified with both glycans, whereas E-cadherin from a poorly-differentiated gastric cancer phenotype (Kato III) mainly exhibited positive reactivity to L-PHA lectin (Supplementary Figure S2A). Despite the reactivity of E-cadherin from MKN28 cells to the Con A lectin (after removal of N-glycans) is significantly higher than E-cadherin from Kato III cells (Figure 2D), no significant differences were verified regarding the E-cadherin *N*-glycosylation mediated by GnT-V (detected by reactivity to the L-PHA lectin) in these two cell lines.

Afterwards, we evaluated the influence of *POMT2* silencing or overexpression on the levels of β1,6 GlcNAc branched *N*-glycans of E-cadherin. *POMT2* knockdown in MKN28 cells resulted in a significantly increased expression of β1,6 GlcNAc branched *N*-glycans on E-cadherin (Figure 5B), although a slight decrease of *MGAT5* transcripts was observed (Supplementary Figure S2B). Conversely, the overexpression of *POMT2* in Kato III cells led to a significant decrease of *MGAT5* mRNA expression (Supplementary Figure S2C), but no significant differences in β1,6 branched *N*-glycans on E-cadherin were found (Figure 5C).

In addition, and to further clarify this potential interplay between branched *N*-glycans and *O*-mannosyl glycans on E-cadherin we used the MKN45 gastric cancer cell line stably transfected with *MGAT5* [18, 41]. According to Figure 5D, overexpression of *MGAT5* leads to a significantly increased modification of E-cadherin with GnT-V-mediated branched *N*-glycans, concomitantly with a significant decrease of *O*-mannosyl glycans. No significant differences were observed on *POMT2* mRNA expression after *MGAT5* overexpression (Supplementary Figure S2D). Taken together, these results suggest that, despite no strict correlation between mRNA levels of *POMT2* and *MGAT5*, there is an inverse relationship between E-cadherin *N*-glycosylation mediated by GnT-V and *O*-mannosylation that appears to occur at the E-cadherin post-translational level.

### Impact of site-specific *N*-glycosylation on E-cadherin *O*-mannosylation

Recently, we demonstrated that among the four potential *N*-glycosylation sites of E-cadherin, Asn-554 is the selected site modified with β1,6 GlcNAc branched *N*-glycans resulting in deleterious effects on E-cadherin functions in cancer [21]. Moreover, we also reported that preventing this aberrant glycosylation mediated by GnT-V at Asn-554 resulted in a recovery of E-cadherin biological functions in cancer [21]. In order to evaluate the relationship between E-cadherin *O*-mannosylation and E-cadherin site-specific *N*-glycosylation, we analyzed the expression of *O*-mannosyl glycans on the different E-cadherin *N*-glycoforms: E-cadherin wild-type (WT), E-cadherin M1 (with Asn-554 abrogated) and E-cadherin M234 (with Asn-566, Asn-618 and Asn-633 abrogated) [21]. According to Figure 5E, E-cadherin M1 exhibited increased modification with *O*-mannosyl glycans, as detected by the higher reactivity to the Con A lectin (after removal of *N*-glycans) when compared to E-cadherin WT and E-cadherin M234. These results strongly support that deletion of Asn-554 (E-cadherin M1), which precludes the addition of β1,6 GlcNAc branched *N*-glycans at this specific site, is associated with an increased modification of E-cadherin with *O*-mannosyl glycans. Additionally, our results further showed that removal of *N*-glycans by *PNGase F* treatment leads to a lower mobility shift of E-cadherin WT and M1 band comparing with E-cadherin M234. These results indicated that these E-cadherin *N*-glycoforms may undergo further glycosylation modification while E-cadherin M234 does not, which support the results regarding Con A lectin blotting.

### Inverse relationship between GnT-V-mediated *N*-glycosylation and *O*-mannosylation in the stomach of transgenic mice and primary human gastric carcinomas

In order to assess *in vivo* the biological relevance of GnT-V-mediated *N*-glycosylation/ *O*-mannosylation interplay we analyzed the expression of β1,6 GlcNAc branched *N*-glycans and *O*-mannosylated proteins in stomach paraffin samples from *MGAT5* transgenic mice models and human gastric carcinomas clinical samples.

**Figure 6:**
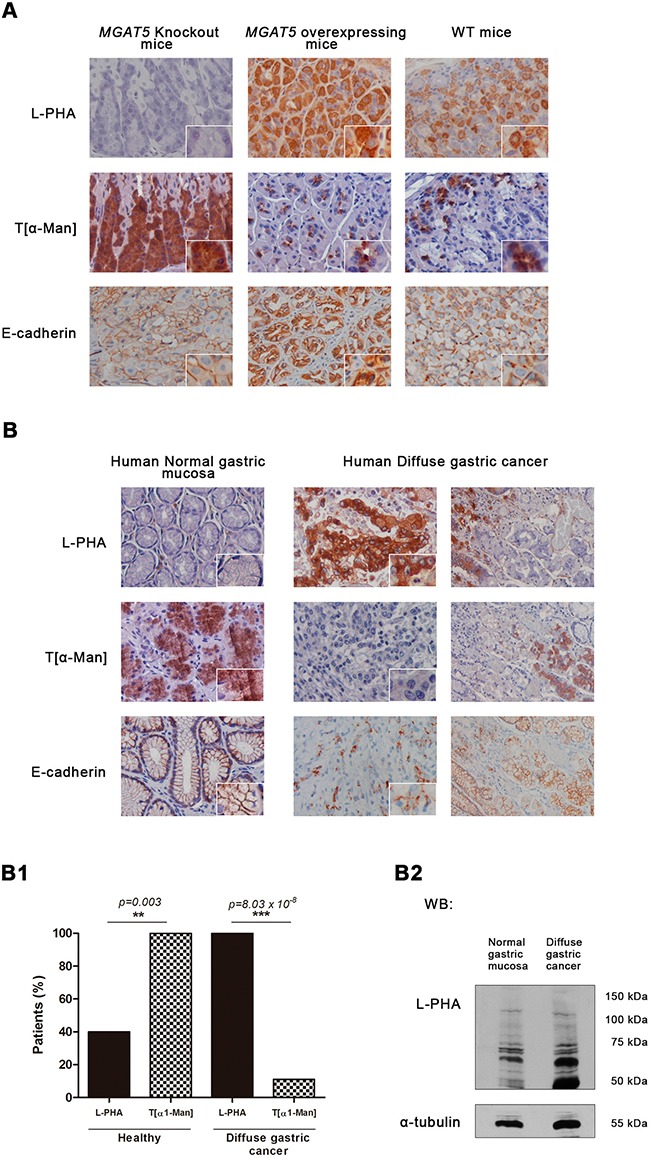
Interplay between GnT-V – mediated N-glycosylation and *O*-mannosylation *in vivo* Expression of β1,6 GlcNAc branched *N*-glycans and *O*-mannosyl glycans in the gastric mucosa of *MGAT5* knockout (KO) and *MGAT5* overexpressing mice, and in human normal gastric mucosa versus diffuse gastric carcinoma. **A.** Histochemical analysis detecting β1,6 GlcNAc branched *N*-glycans showed an absent, a strong, and a moderate L-PHA lectin staining in the gastric mucosa of *MGAT5* KO, *MGAT5* transgenic, and WT mice, respectively. Concerning the T[α-Man] immunoreactivity, gastric mucosa from WT and *MGAT5* transgenic mice showed low expression of *O*-mannosylated proteins. In contrast, a overexpression of O-mannosylated glycoproteins is verified in *MGAT5* KO gastric mucosa. **B.** Interplay between GnT-V-mediated glycosylation and protein *O*-mannosylation in human gastric mucosa. L-PHA histochemistry showed almost no reactivity in the human normal gastric mucosa and a marked positivity in neoplastic cells from diffuse gastric carcinoma. Regarding the expression of *O*-mannosylated proteins, we verified that neoplastic cells from diffuse gastric carcinoma positive to L-PHA lectin and showing an aberrant expression of E-cadherin at the cytoplasm, exhibited no reactivity to T[α-Man] mAb. In contrast, cells from normal adjacent mucosa and human normal gastric mucosa characterized by a cell membrane localization of E-cadherin and almost no expression of β1,6 GlcNAc branched *N*-glycans, were positive to T[α-Man] mAb. Original magnification: 400x **(B1)** Evaluation of *O*-mannosyl glycans/ β1,6 GlcNAc branched glycans interplay (*n*=10 healthy patients; *n*= 18 diffuse gastric carcinoma patients). **(B2)** β1,6 GlcNAc branched glycans expression in total tissue lysate from normal gastric mucosa and diffuse gastric carcinoma. An increased expression of β1,6 GlcNAc branched *N*-glycans detected by L-PHA lectin blotting was verified in total tissue lysate from diffuse gastric carcinoma. Blot represents two independent biological experiments.

Previously, we have demonstrated that GnT-V-mediated glycosylation was associated with an abnormal pattern of E-cadherin expression in the gastric mucosa of GnT-V transgenic mice [21]. Thus, we herein evaluated the correlation between the expression of GnT-V-mediated glycosylation and *O*-mannosylation in the gastric mucosa of *MGAT5* knockout (KO) and *MGAT5* overexpressing mice comparing with wild-type (Figure 6A). The results showed that gastric mucosa of *MGAT5* KO, characterized by no reactivity to L-PHA lectin (no expression of β1,6 GlcNAc branched *N*-glycans) and displaying a normal E-cadherin distribution in the basolateral cell membrane, showed overexpression of *O*-mannosylated proteins. On the other hand, mice overexpressing *MGAT5* with a strong staining with L-PHA lectin and an aberrant pattern of E-cadherin expression, displayed reduced levels of O-mannosylated proteins. These results indicated that *MGAT5* expression affects the protein *O*-mannosylation.

In human gastric cancer context, gastric mucosa from healthy patients consistently displays positive staining to the T[α1-Man] mAb that is accompanied with very low levels (<10%) of β1,6 GlcNAc branched *N*-glycans (as detected with L-PHA lectin) (Figure 6B-B1). On the other hand, neoplastic cells with an abnormal pattern of E-cadherin expression, showed high levels of β1,6 GlcNAc-branched *N*-glycans concomitantly with negligible expression of *O*-mannosylated proteins (*p value*=0.03). Cells from normal adjacent mucosa with a cell membrane localization of E-cadherin exhibited no reactivity to L-PHA together with positivity to T[α-Man] mAb. Additionally, L-PHA lectin blotting represented in Figure 6B2 demonstrated higher expression of β1,6 GlcNAc branched *N*-glycans on diffuse gastric cancer lysates, when compared with normal gastric mucosa. These results strongly indicated the existence of a significant inverse correlation between protein *O*-mannosylation and β1,6 GlcNAc branched *N*-glycans that accompany gastric carcinogenesis. (Figure 6B1).

Overall, these results support the existence of biological interplay between GnT-V-mediated glycosylation and *O*-mannosylation affecting the functions of a key tumor suppressor protein in diffuse gastric carcinogenesis, E-cadherin.

## DISCUSSION

Dysregulation of E-cadherin is a molecular signature of epithelial cancer progression and glycosylation post-translational modifications are a fundamental mechanism described to regulate E-cadherin functions [12]. The molecular mechanisms that regulate E-cadherin functions in cancer through the newly described *O*-mannosyl glycans is far from being elucidated [35]. Moreover, the potential interplay between the two major forms of E-cadherin post-translational modifications, *N*-glycosylation and *O*-mannosylation, and its relevance in cancer biology is completely unknown.

In this study we have demonstrated that protein *O*-mannosylation in human gastric cancer patients is overall reduced, suggesting that the *O*-mannosylation profile of several glycoproteins are changed affecting thereby the cellular regulation. Particularly, we found that E-cadherin undergoes a decreased *O*-mannosylation in cancer that resulted in the impairment of E-cadherin functions in cancer cells through interfering in its cell membrane localization and in the assembly and competence of adherens junctions. A novel functional link between the dysregulation of the tumour suppressor protein E-cadherin and the reduced expression of *O*-mannosyl glycans is thus established. Interestingly, when *O*-mannosylation was promoted in a gastric cancer context, a recovery of E-cadherin membrane localization together with an increased stability of the intercellular adhesive complex was observed.

We also provide new evidence for the existence of a yet uncovered interplay between *O*-mannosylation and *N*-glycosylation in human gastric carcinogenesis that appears to operate in a coordinated and site-specific manner on E-cadherin glycoprotein. GnT-V-mediated branched *N*-glycosylation has been described to contribute to tumour invasiveness and metastasis, being thus an instrumental mechanism of E-cadherin dysregulation in cancer [42–44]. Indeed, modification of E-cadherin with β1,6 GlcNAc branched *N*-glycans leads to E-cadherin cellular mislocalization and an incorrect assembly of adherens-junctions, compromises cell-cell adhesion and downstream signalling pathways [18, 19, 45]. More importantly, the expression of β1,6 GlcNAc branched *N*-glycans on E-cadherin is associated with poor survival rates of gastric cancer patients [21].

Altered POMT2 expression in gastric cancer cells consistently resulted in altered expression of branched complex *N*-glycans on E-cadherin in an inverse and coordinated manner. *POMT2* knockdown resulted in an increased GnT-V-mediated branched *N*-glycans on E-cadherin. Likewise, when the branched *N*-glycosylation pathway is induced, by overexpressing *MGAT5*, a significant decrease of *O*-mannosyl glycans attached to E-cadherin was observed. GnT-V-mediated *N*-glycosylation and *O*-mannosylation processes are thus two crucial inter-players that contribute to the E-cadherin functions.

In a previous work we reported that among the four potential *N*-glycosylation sites of E-cadherin, the Asn-554 is selectively occupied with β1,6-GlcNAc-branched *N*-glycans. Preventing the addition of these branched *N*-glycans at Asn 554 resulted in a protective effect on E-cadherin, precluding its functional dysregulation in cancer [21]. In this study, we demonstrated that presence of the branched *N*-glycans at E-cadherin Asn-554 directly affects its *O*-mannosylation pattern. The prevention of this site-specific glycosylation modification by Asn mutation potentiates the *O*-mannosylation profile of E-cadherin associated with a recover of its biological functions. The exact mechanism behind this coordinated interplay between *O*-mannosylation and complex *N*-glycosylation on E-cadherin needs to be further dissected. The competition for donor mannose substrate in the ER; protein conformational changes; localization of glycosyltranferases in ER and/or Golgi compartments; the physio-pathological context of the tissue/cell may constitute potential explanations that need to be further investigated.

Importantly, this coordinated interplay was also observed *in vivo*, in the stomach of two different *MGAT5* transgenic mouse models. The *MGAT5* knockout mice characterized by no GnT-V-mediated branched glycosylation [46] revealed a remarkable reactivity to *O*-mannosyl glycans. Conversely, *MGAT5* overexpressing and wild-type mice with high to moderate levels of branched *N*-glycans showed reduced levels of *O*-mannosyl glycans. Moreover, and relevant from the clinical point of view, this interplay between the two glycosylation forms was also consistently demonstrated in a series of patients with primary diffuse gastric carcinoma. Gastric neoplastic cells, characterized by an aberrant E-cadherin expression at the cytoplasm, exhibited increased expression of β1,6 GlcNAc branched *N*-glycans concomitantly with negligible levels of *O*-mannosyl glycans. The opposite was observed in normal gastric mucosa. These results strongly suggest that GnT-V-mediated glycosylation negatively affects protein *O*-mannosylation.

**Figure 7:**
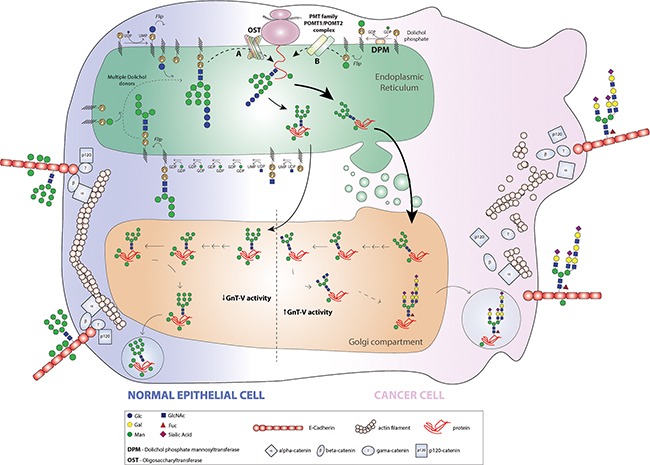
Model for the pattern of E-cadherin *O*-mannosylation and the interplay with GnT-V-mediated *N*-glycan branching in a cancer context In a normal epithelial cell, E-cadherin undergoes a preferential *O*-mannosylation modification contributing to the proper adhesive functions of E-cadherin. In the ER lumen, the mannose residues are added at the E-cadherin EC4 domain by action of POMT2/POMT1 complex, and along the Golgi apparatus E-cadherin does not undergo complex branched *N*-glycosylation mediated by GnT-V. The predominance of *O*-mannosyl glycans rather than complex branched *N*-glycans contributes to a protective effect on E-cadherin. During malignant transformation, both the *POMT2* gene and enzyme are downregulated and consequently the pattern of E-cadherin *O*-mannosylation decreases. Further, and owing to increased *MGAT5* expression and GnT-V activity frequently observed in a cancer cell, E-cadherin is predominantly modified with β1,6 GlcNAc branched *N*-glycans at E-cadherin Asn-554. The presence of these deleterious branched N-glycans structures combined with reduced levels of *O*-mannosyl glycans on EC4 ectodomain lead to loss of E-cadherin suppressive functions in cancer, thus contributing to tumour progression and metastases.

Taken together, our observations support a new mechanism of E-cadherin regulation in cancer cell biology. In a normal epithelial cell phenotype, E-cadherin undergoes a preferential *O*-mannosylation modification, which contributes to the proper adhesive functions of E-cadherin. In the ER lumen, POMT2/POMT1 complex catalyses the addition of mannose residues at the E-cadherin EC4 domain and along the Golgi apparatus E-cadherin does not undergo complex branched *N*-glycosylation mediated by GnT-V. The predominance of *O*-mannosyl glycans rather than complex branched *N*-glycans contribute to a protective effect on E-cadherin, precluding its functional dysregulation (Figure 7). During malignant transformation, the balance *O*-mannosyl glycans/ complex branched *N*-glycans in E-cadherin is altered. In a cancer cell, both the *POMT2* gene and enzyme are downregulated and consequently the pattern of E-cadherin *O*-mannosylation decreases. Further, and owing to increased *MGAT5* expression and GnT-V activity frequently observed in a cancer cell, E-cadherin is predominantly targeted by GnT-V-mediated glycosylation at the Golgi apparatus, where β1,6-GlcNAc-branched N-glycans are added to Asn-554 of E-cadherin. The presence of these deleterious branched *N*-glycans structures together with reduced levels of *O*-mannosyl glycans on EC4 ectodomain lead to loss of E-cadherin suppressive functions in cancer, thus contributing to tumour progression and metastases (Figure 7).

Overall, this study proposes a newly identified mechanism of E-cadherin (dys)regulation in cancer that is precisely coordinated through the interplay between *O*-mannosylation and *N*-glycosylation machinery that operates at the protein site-specific level Moreover, these differential patterns of E-cadherin glycosylation can constitute promising (glyco)biomarkers for improving gastric cancer patients' clinical management.

## MATERIALS AND METHODS

### Tissue immunohistochemistry and lectin staining

Formalin-fixed paraffin-embedded tissue from human normal gastric mucosa (*n*=10), human diffuse gastric cancer (*n*=18); both obtained from the S. João Centre Hospital, Porto, Portugal); *MGAT5* knockout and *MGAT5* transgenic mouse stomach (n=3; C57BL/6 background; kindly provided by Michael Pierce' and Eiji Miyoshi' labs, respectively) were used for E-cadherin and L-PHA staining as previously described [18]. For immunoexpression of O-mannosylated protein, the sections were incubated with T[α1-Man]- specific monoclonal antibody (dilution 1:50) for one hour at RT (unpublished results). This study was approved by the ethical committee of Centro S. João Centre Hospital, Porto, Portugal and an informed consent was obtained for all the subjects.

### Cell lines

Human gastric carcinoma cell lines MKN28, Kato III, and AGS cells (transfected with E-cadherin *N*-glycosylation site mutant, as previously described [21]) were grown in RPMI 1640 GlutaMAX supplemented with 10% fetal bovine serum (Gibco, Invitrogen) and 1% penicillin-streptomycin (Gibco, Invitrogen), and maintained at 37 °C in an atmosphere of 5% CO_2_. MKN45 gastric cell line stably transfected with human GnT-V and with the empty vector (mock cells) were cultured in the previous conditions under the selection of G418 (500 μg/mL) [41, 47, 48].

### Cell transfection and siRNA transfection

Kato III suspension cells were transfected with plasmid pTW49 [49] using Lipofectamine 2000 (Invitrogen), according to the manufacturer's instructions. siRNA targeting *POMT2* (ON-TARGETplus Human POMT2siRNA-SMARTpool) and control siRNA (ON-TARGETplus Non-targeting pool) were purchased from Dharmacon. siPOMT2 (0-200 nM) was transfected into MKN28 cell line with Lipofectamine 2000 (Invitrogen) according to the manufacturer's instructions. The efficiency of *POMT2* knockdown was evaluated at RNA and protein level by quantitative Real Time- PCR (qRT-PCR) and western blot, respectively. *POMT2* knockdown was optimal with 80 nM of siRNA after 48h.

### RNA expression and quantification

Total RNA was extracted from cell lines using TRI reagent (Sigma-Aldrich), according to manufacturer's protocol. RNA yield and quality were determined spectophotometrically and 1000 ng of total RNA were reverse transcribed to single stranded cDNA using Superscript II Reverse Transcriptase and random hexamer primers (Invitrogen, Oregon, USA). qRT-PCR was carried out in triplicates using source RNA from three biological replicas, for the target genes *POMT2*, *POMT1* and for the endogenous controls 18S and GAPDH. Data were analyzed by the ΔΔCT method [50].

### Immunoblotting, lectin blotting

Protein lysates (20 μg) were subjected to 7.5% SDS-PAGE, transferred to nitrocellulose membranes and probed with primary antibodies: β-actin/ α-tubulin (previously described [18]) and polyclonal rabbit anti-POMT2 (1:500; polyclonal serum directed against the ER-lumenal loop5-domain Val373-Leu470) [49]

To evaluate the expression of *O*-mannosyl glycans on E-cadherin, E-cadherin was immunoprecipitated [18] and digested with *PNGase F* (according to manufacturer's protocol; New England, Biolabs) to remove *N*-glyans (overnight, 37°C) followed by lectin blotting using Biotinylated Concanavalin A (Con A) (1-5 μg/mL; Vector Laboratories). The efficiency of *PNGase F* was evaluated by comparing the mobility shift of E-cadherin from MKN28 and Kato III cell lines with E-cadherin N-glycoform M1234 naked (E-cadherin mutant with all potential *N*-glycosylation sites mutated and thus lacking N-glycans structures) (Supplementary Figure S3A-S3B). Galanthus nivalis lectin (GNA) was also used to evaluate the *O*-mannosylation profile of E-cadherin after removal of N-glycans by *PNGaseF* treatment (data not shown).

To evaluate O-GlcNAc expression on E-cadherin, E-cadherin immunoprecipitated was subjected to *PNGase F* digestion, and the membranes were probed with wheat germ agglutinin (WGA) lectin (10 μg/mL; Vector Laboratories). Mucin-type O-glycosylation (STn) of E-cadherin was evaluated by using the monoclonal antibody TKH2 (mouse IgG1). Expression of β1,6 GlcNAc branched N-glycans analysis on E-cadherin, was assessed by probing the membranes with L-PHA lectin (as previously described [18]). AGS cell line that endogenously lacks E-cadherin expression was used as a negative control for the E-cadherin immunoprecipitations (Supplementary Figure S3C).

### Immunofluorescence

Cells were grown on six-well plates with coverslip, fixed with methanol and blocked with bovine serum albumin (BSA) 5% in 1x PBS. Cells were then incubated with mouse anti-E-cadherin mAb (BD Bioscience; 1:200 diluted in BSA 5%, one hour at RT), and then with Alexa Fluor 488 anti-mouse (Invitrogen; 1:500 diluted in BSA 5%; one hour of incubation in the dark) [18]. Immunofluorescent images were obtained using a Zeiss Imager.Z1 AxioCam MRm (Carl Zeiss, Jena, Germany).

### Slow-aggregation assay

Wells of a 96-well-plate were coated with 50 μl of an agar (0.66% w/v; Sigma) solution. Cells (1.25 × 10^4^ per well) were seeded onto the agar gel in a 96-well plate. Experiments were carried out in triplicate.

### Statistics

Statistical analyses were performed using the Graph Pad program (GrapPad Software, Inc., La Jola, CA, USA). Student's tests were used to calculate the significance in an interval of 95% confidence levels. Values of P ≤ 0.05 were considered to be statistically significant (Student's t-test: *P≤0.05; **P≤0.01; ***P≤0.001).

## SUPPLEMENTARY FIGURES


