# Supplementary material for: O-mannosylation and N-glycosylation: two coordinated mechanisms regulating the tumour suppressor functions of E-cadherin in cancer
Source: Oncotarget. 2016 Aug 12;7(40):65231–46. doi: 10.18632/oncotarget.11245 (PMC5323151; doi:10.18632/oncotarget.11245)
Supplement: Supplementary file 1 [file oncotarget-07-65231-s001.pdf]

O–mannosylation and *N*-glycosylation: two coordinated mechanisms regulating the tumour suppressor functions of E-cadherin in cancer

SUPPLEMENTARY FIGURES

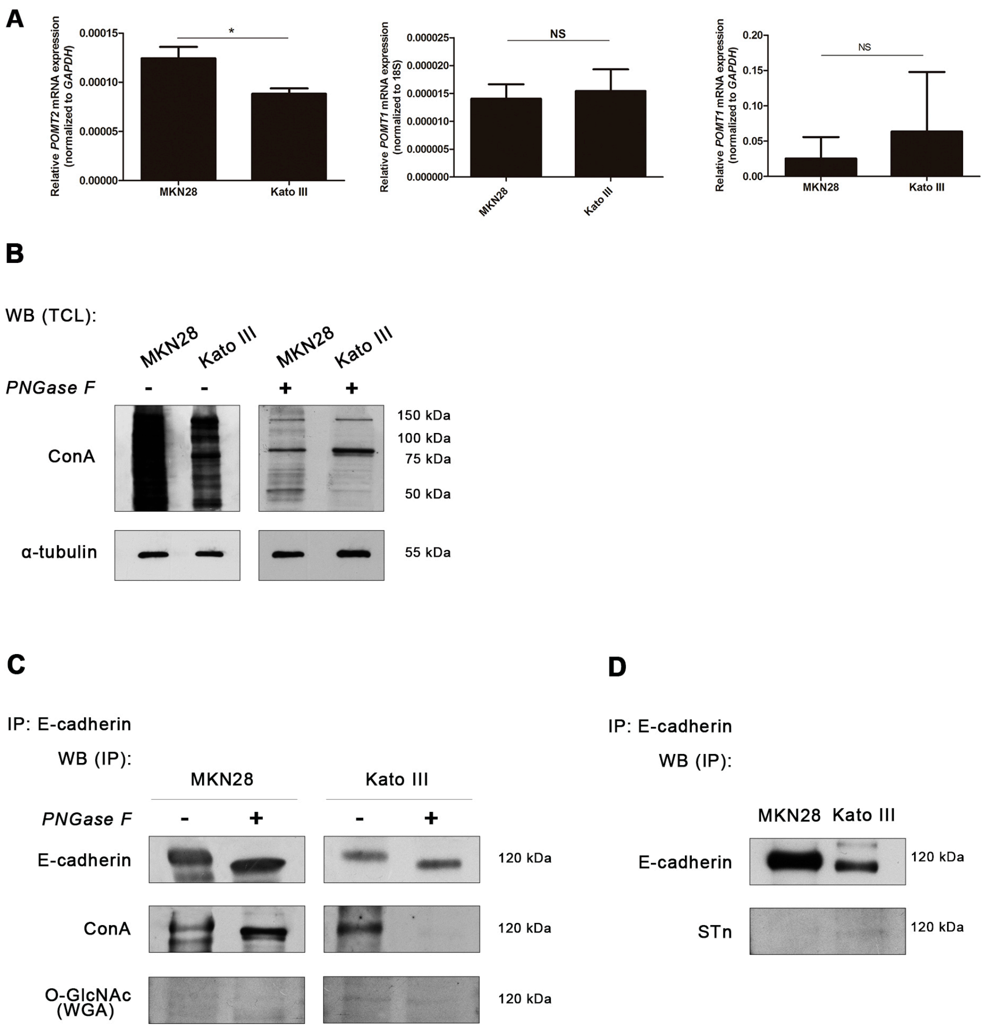

Supplementary Figure S1: Expression of *O*-mannosyl glycans and specifically on E-cadherin in MKN28 and Kato III cells. A. *POMT1* mRNA expression. No significant differences were found in the transcript levels of *POMT1* in MKN28 and Kato III cells. Same results of *POMT1* and *POMT2* mRNA transcripts were obtained using another endogenous control GAPDH. Results are described as mean ± s.d of three independent biological replicates **B.** *O*-mannosyl glycans profile. *N*-linked oligosaccharides containing α-linked mannose residues in their core region were removed from total cell lysates by *PNGase F* digestion. Con A- mannose-binding lectin blotting was performed to evaluate the expression of *O*-mannosyl glycans from glycoproteins. The reactivity to Con A lectin decreased after removal of *N*-glycans. Note that both represented lectin blots corresponded to the same exposure time. **C.** O-mannosylation and O-GlcNAcylation of E-cadherin in MKN28 and Kato III cell lines. E-cadherin immunoprecipitated from MKN28 cells exhibited a positive reactivity to Con A lectin (with and without *PNGase F* treatment). Regarding Kato III cells, E-cadherin immunoprecipitated showed a significant lower reactivity to Con A lectin after removal of N-glycans. Note that both represented lectin blots corresponded to the same exposure time. Regarding O-GlcNAcylation, E-cadherin immunoprecipitated from Kato III cells exhibited a residual positivity to WGA lectin after *PNGaseF* digestion which may suggest expression of O-GlcNAc residue on E-cadherin **D.** Mucin-type *O*-glycosylation of E-cadherin in MKN28 and Kato III cell lines. The faint positivity of E-cadherin from Kato III cell line to TKH2 mAb may suggest residual expression of STn on E-cadherin.

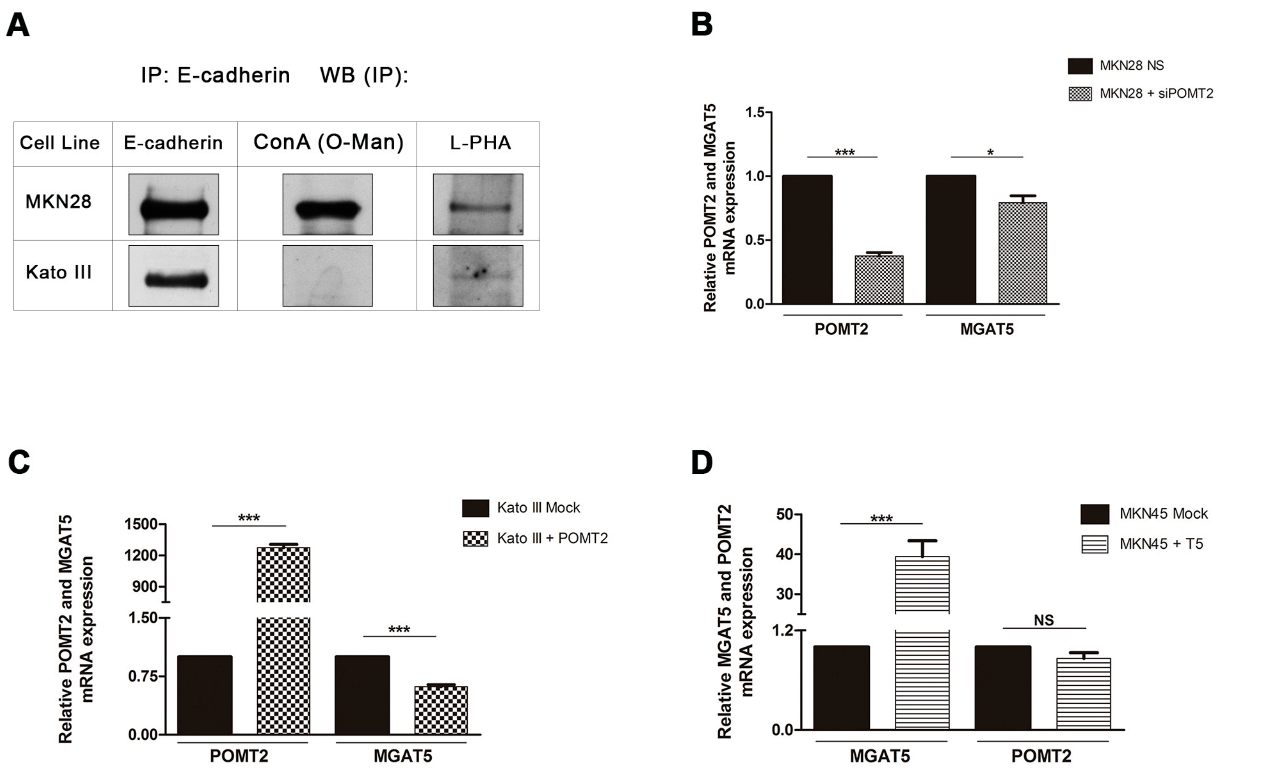

Supplementary Figure S2: A. Reactivity of E-cadherin immunoprecipitated from MKN28 and Kato III cells to the lectins Con A (after removal of *N*-glycans) and L-PHA. **B-D.** Evaluation of mRNA transcripts of *POMT2* and *MGAT5* in three different cell backgrounds: MKN28 and Kato III cell lines after modulation of *POMT2* expression either by (B) knockdown or by (C) overexpression, and (D) after overexpression of *MGAT5* in MKN45 cell line. The relative *POMT2* and *MGAT5* mRNA expression of MKN28 + siPOMT2, Kato III + POMT2 and MKN45 + T5 cells are expressed as the fold increase, compared with NS cells or mock cells, respectively, which was taken as 1. The data presented is referred to three independent biological replicates.

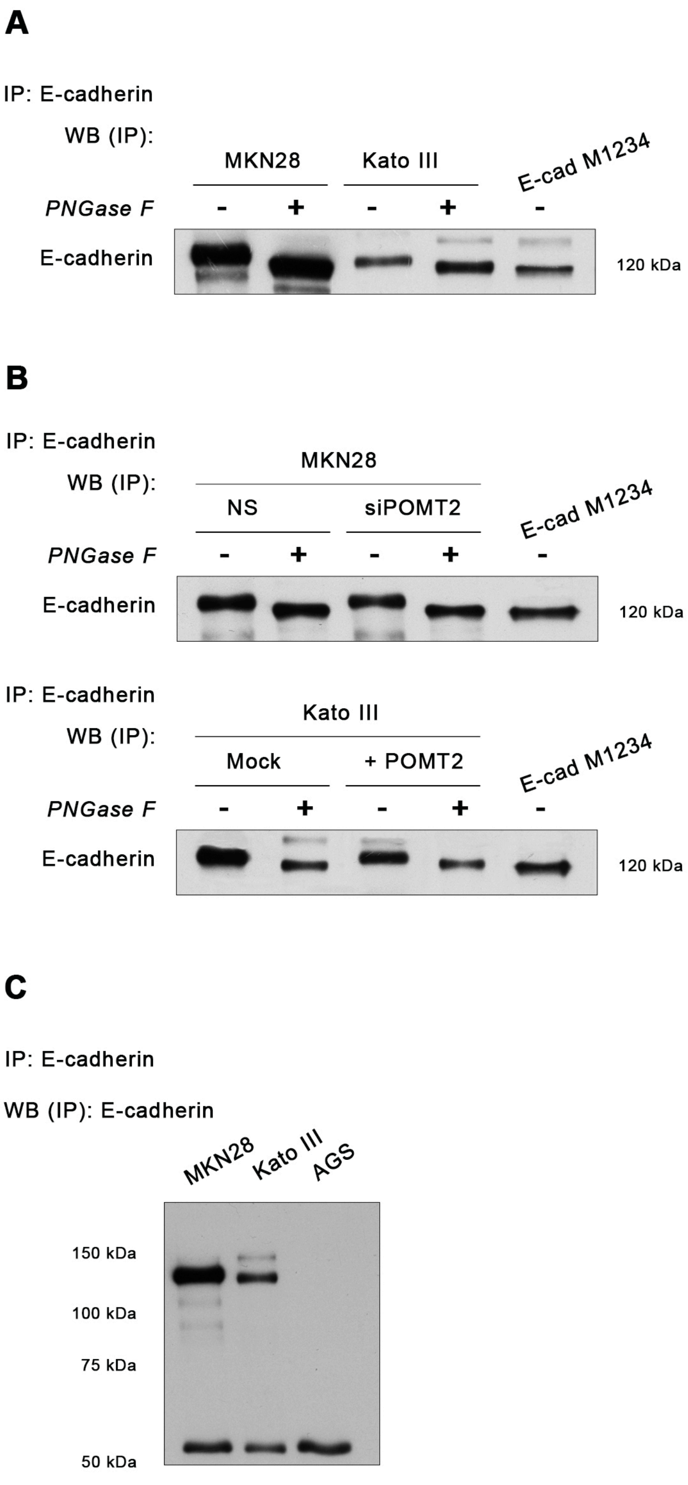

Supplementary Figure S3: Evaluation of *PNGase F* efficiency by mobility shift of E-cadherin. E-cadherin immunoprecipitated from **A.** MKN28 and Kato III cell lines **B.** after POMT2 knockdown in MKN28 or POMT2 overexpression in Kato III cells was treated with *PNGase F* to release the *N*-glycans. The mobility shift resulting from this treatment was compared with E-cadherin *N*-glycan naked (E-cadherin M1234, with all the potential *N*-glycosylation sites mutated, lacking *N*-glycans structures). **C.** E-cadherin immunoprecipitation from MKN28, Kato III and AGS cell lines. AGS cell lines was used as a biological control for the E-cadherin immunoprecipitation experiments followed by immunoblotting to E-cadherin, β-catenin, p120-catenin or lectins blotting.
